# Algae-based oral recombinant vaccines

**DOI:** 10.3389/fmicb.2014.00060

**Published:** 2014-02-17

**Authors:** Elizabeth A. Specht, Stephen P. Mayfield

**Affiliations:** California Center for Algae Biotechnology, University of California at San DiegoLa Jolla, CA, USA

**Keywords:** oral vaccines, recombinant subunit vaccines, microalgae, plant-produced vaccines, algal engineering

## Abstract

Recombinant subunit vaccines are some of the safest and most effective vaccines available, but their high cost and the requirement of advanced medical infrastructure for administration make them impractical for many developing world diseases. Plant-based vaccines have shifted that paradigm by paving the way for recombinant vaccine production at agricultural scale using an edible host. However, enthusiasm for “molecular pharming” in food crops has waned in the last decade due to difficulty in developing transgenic crop plants and concerns of contaminating the food supply. Microalgae could be poised to become the next candidate in recombinant subunit vaccine production, as they present several advantages over terrestrial crop plant-based platforms including scalable and contained growth, rapid transformation, easily obtained stable cell lines, and consistent transgene expression levels. Algae have been shown to accumulate and properly fold several vaccine antigens, and efforts are underway to create recombinant algal fusion proteins that can enhance antigenicity for effective orally delivered vaccines. These approaches have the potential to revolutionize the way subunit vaccines are made and delivered – from costly parenteral administration of purified protein, to an inexpensive oral algae tablet with effective mucosal and systemic immune reactivity.

## INTRODUCTION

Infectious diseases directly account for nearly 25% of deaths worldwide, and are a predominant cause of morbidity and mortality in the developing world (Fauci et al., 2005). Even for diseases for which vaccines exist, limited access – due to financial as well as infrastructural or medical personnel limitations – is a major contributor to this high infectious disease burden. Many developing world diseases do not yet have vaccines, in part because traditional vaccine production costs present a significant investment hurdle, considering the financial capacity of the intended consumers. Both cost and ease of administration are challenges that must be tackled to address this undue burden on global health and productivity.

Oral vaccination has many distinct advantages over parenteral administration, but has proven difficult to achieve thus far, reflected by the scarcity of licensed oral vaccines. Perhaps the most significant benefit of oral vaccination is the ability to elicit both mucosal and systemic immunity. As most human pathogens enter via mucosal surfaces – either nasally, orally, or by sexual transmission – mucosal immunity can serve as a first line of defense to prevent infection before it reaches the bloodstream (Mason and Herbst-Kralovetz, 2012). Oral vaccines also obviate the need for trained medical personnel to administer them and reduce the risks of infection associated with needles. They also have higher compliance from patients, owing to the lack of fear and resistance associated with injections. Both of these latter aspects are important considerations for successful vaccination campaign coverage in remote or resource-limited settings.

Plant-produced vaccines have two critical advantages: much lower cost than traditional recombinant vaccine platforms, and improved safety because of insusceptibility to mammalian pathogen contamination. The batch costs of plant-produced vaccines may be as much as a thousand times less than traditional animal cell culture or even bacterial or yeast cell culture, though it has been noted that this will not translate directly to per-dose cost because downstream sales, packaging, and distribution costs are similar regardless of production method (Rybicki, 2009). The current status of plant-produced vaccines in pre-clinical and early phase human clinical trials has been extensively reviewed (Lossl and Waheed, 2011; Mason and Herbst-Kralovetz, 2012; Rosales-Mendoza et al., 2012a,b; Guan et al., 2013; Jacob et al., 2013); despite positive preliminary data, none have made it through to licensing. The only licensed plant-produced vaccine is a veterinary injectable vaccine against Newcastle disease virus in poultry, made from purified antigen expressed in cultured tobacco cells. Dow AgroSciences received Food and Drug Administration (FDA) approval for the vaccine in 2006, but only as a demonstration that plant-produced vaccines can meet the stringent regulatory requirements for approval; it is not currently for sale (Rybicki, 2009).

Plant cells are of particular interest for oral vaccines because their rigid cell walls provide exceptional antigen protection through the stomach into the intestines, where they can access the gut-associated lymphoid tissue (Kwon et al., 2013). Expression within chloroplasts or other storage organelles may also provide additional protection (Khan et al., 2012). While vaccine antigens have been transformed into many edible species including lettuce, tomato, potato, and tobacco, expression in stable transformed crop plants has suffered from low yields, typically less than 1% of total soluble protein (TSP; Lossl and Waheed, 2011). Yields have been increased by transient expression using recombinant viral vectors or *Agrobacterium* infection, but this expression is typically unstable (Rybicki, 2009). Even using these strategies, the most consistently high-yielding host species is tobacco, which is inedible and therefore would require purification prior to vaccine administration (Lossl and Waheed, 2011).

### ALGAE AS A RECOMBINANT PROTEIN PRODUCTION PLATFORM

Green microalgae have proven to be highly useful protein production platforms for a variety of industrial and therapeutic applications, particularly for complex or heavily disulfide-bonded proteins. The chloroplast provides a unique enclosed compartment that facilitates folding (Chebolu and Daniell, 2009), and transgene products have been shown to accumulate to high levels in the algal chloroplast – as high as 10% of TSP (Manuell et al., 2007; Surzycki et al., 2009). Unlike prokaryotes, chloroplasts of algae contain much of the same sophisticated cellular folding machinery as other eukaryotic organisms like yeast. While the algal nuclear genome can also be transformed, to date most transgene expression has been from the chloroplast genome due to reduced gene silencing and higher protein accumulation.

The green alga model organism *Chlamydomonas reinhardtii* has been used to produce a number of human and animal therapeutically relevant proteins, including full-length human antibodies (Tran et al., 2009), signaling molecules such as vascular endothelial growth factor (Rasala et al., 2010), and structural proteins like fibronectin (Rasala et al., 2010). Though expression levels are highly variable by gene, improvements in codon optimization (Franklin et al., 2002; Surzycki et al., 2009) and characterization of ideal gene regulatory elements (Rasala et al., 2011; Specht and Mayfield, 2013) continue to increase levels of transgene expression. *C. reinhardtii*’s success and future potential as a therapeutic protein production platform has been recently reviewed (Rasala and Mayfield, 2011).

### ADVANTAGES OF AN ALGAL VACCINE PRODUCTION HOST

Unicellular green algae possess all the positive attributes of plant systems, plus several unique advantages over terrestrial plants as vaccine production hosts. Algal biomass accumulation is extremely rapid, and the entirety of the biomass can be utilized for vaccine production, unlike plants that expend energy producing supporting tissues that do not contain the vaccine antigen or cannot be harvested easily. Algae are also not restricted by growing season or local soil fertility, and concerns of cross-contamination of nearby food crops are non-existent. Enclosed bioreactors can be used for higher biomass yields and to reduce concerns of environmental escape (Franconi et al., 2010), and media can be recycled to minimize water and nutrient loss. The 2002 discovery of transgenic viral capsid protein-expressing maize in food harvests of nearby corn and soybean crops effectively halted efforts to produce vaccines in edible crop plants, making a food crop-based oral vaccine highly unlikely (Rybicki, 2009). Green algae such as *C. reinhardtii* are generally recognized as safe (GRAS) by the FDA, resurrecting hope that unprocessed edible vaccines can be produced in a photosynthetic organism.

Crop plants can contain hundreds of chloroplasts per cell, and each chloroplast harbors dozens of copies of its plastid genome. In contrast, *C. reinhardtii* contains a single chloroplast that occupies about half of the volume of the cell (Franklin and Mayfield, 2005), making stable homoplasmic transformed lines much easier to obtain (a few weeks versus several months) and allowing for increased yields of plastid-expressed vaccine antigens, which account for nearly all antigens expressed to date in algae. This genomic stability, combined with the ability to tightly regulate growth conditions inside contained bioreactors, allows for more consistent expression levels than terrestrial plants, which can vary by several-fold.

Finally, algae can be easily preserved by lyophilization, and two studies of algal-produced vaccine antigens have verified that dried algae stored at room temperature for 6 months (Gregory et al., 2013) or even 20 months (Dreesen et al., 2010) exhibit nearly equivalent antigen effectiveness as freshly harvested algae, though storage at 37° did begin to cause a loss of activity over time (Gregory et al., 2013). The algal cell wall appears sufficient to withstand harsh conditions within the stomach, as very little antigen degradation was observed after whole cells were incubated with pepsin at pH 1.7 (Dreesen et al., 2010). These observations indicate that algae are an ideal host for vaccine transport without cold-chain supply, and that the cells provide adequate protection for antigens en route to the intestinal mucosal lymph tissue, obviating the additional expense associated with encapsulation.

### ALGAL VACCINE PROGRESS

The first reported algal-synthesized vaccine antigen was a chimeric molecule comprising the foot-and-mouth disease virus structural protein VP1 and the beta subunit of cholera toxin (CTB), a known mucosal adjuvant (Sun et al., 2003). This antigen had been previously expressed in plants and had demonstrated oral immunity in mice (Wigdorovitz et al., 1999), but advancement of trials was hindered by low expression levels. In *C. reinhardtii*, 3–4% TSP was reported, but higher yields may be possible because the strains examined were not completely homoplasmic (Sun et al., 2003).

The next report of an algal-produced vaccine antigen showed the first *in vivo* data for efficacy conferring immunity. The classical swine fever virus (CSFV) surface protein E2 was expressed from the *C. reinhardtii* chloroplast genome, and total protein extracts were administered subcutaneously with Freud’s adjuvant or orally by gavage with no adjuvant. Subcutaneous immunization reportedly induced a significant immune response, but no data for this result was shown. No systemic or mucosal immune response was detected after the oral immunization, and it was suggested that a mucosal adjuvant may be necessary for oral administration to be effective (He et al., 2007).

Wang et al. (2008) expressed the human glutamic acid decarboxylase, a known Type 1 diabetes autoimmune antigen, which reacted with sera from non-obese diabetic mice. Surprisingly, detectable expression was achieved using a non-codon-optimized gene. A more thorough investigation of the factors affecting vaccine antigen expression in algae found that indeed codon optimization is critical for high yield. It has also been noted that yield is highly variable among individual transformants despite the fact that chloroplast transformation proceeds by homologous recombination, eliminating positional effects within the genome (Surzycki et al., 2009).

Oral immunization was finally shown to be effective when the antigen of interest was fused to the B subunit of CTB, which forms a pentameric structure and binds the GM1 ganglioside for internalization into intestinal cells. After feeding freeze-dried algae repeatedly to mice, fecal IgA and systemic IgG antibody titers reached similarly high levels for both the intended *Staphylococcus aureus* antigen and CTB. Significantly, within a week of finishing the 5-week oral vaccination, 80% of immunized mice survived a lethal challenge with *S. aureus* that killed all control mice within 48 h (Dreesen et al., 2010).

Two studies earlier this year reported relatively low yields of two additional algal-produced antigens, but they are still promising compared to previous literature using alternative systems. A human papillomavirus E7 protein, while only accumulated to 0.12% TSP, expressed similar to or better than in other plant systems and did not require fusion to a stabilizing protein to achieve consistent expression. Furthermore, the algal chloroplast-produced E7 was soluble, whereas the plant-produced E7 was found predominantly in the insoluble fraction using multiple solubilization buffers. While the antibody titer elicited by affinity purified protein was much higher, a crude algal extract was shown to be equally effective at preventing tumor development and promoting mouse survival (Demurtas et al., 2013). A chimeric antigen intended to prevent hypertension, consisting of a fusion between angiotensin and a Hepatitis B antigen as a carrier, was the first algal vaccine to be expressed from the nuclear genome without chloroplast targeting. While it only accumulated to 0.05% TSP, it was detectable by Western blot from algal TSP extracts (Soria-Guerra et al., 2014).

Since 2010, several studies have shown that malarial transmission-blocking vaccines can be produced in *C. reinhardtii*. Transmission-blocking vaccines target surface proteins that appear on the sexual and gamete stages of *Plasmodium*, the causative pathogen of malaria. There is some evidence that these vaccines may provide partial protection to individuals, but the main benefit of vaccination with a transmission-blocking vaccine is derived from herd immunity preventing the spread of the disease. Therefore, it is especially critical that transmission-blocking vaccines can be delivered easily and at extremely low cost, to reach threshold coverage of the huge populations living in malaria-endemic regions. One difficulty of producing these *Plasmodium *surface proteins is that they contain multiple EGF-like domains that are heavily disulfide-bonded, rendering them difficult to fold and therefore difficult to accumulate to high levels without forming insoluble aggregates (Gregory et al., 2012). Interestingly, *Plasmodia* appear to not glycosylate their proteins (Gowda and Davidson, 1999), making algal chloroplasts suitable hosts as the chloroplast also does not contain glycosylation machinery.

A total of six algae-produced malarial antigens or fragments thereof – *Pfs*25, *Pfs*28, *Pfs*48/45, *Pf*MSP1, *Pb*MSP1, and *Pb*AMA1 – have been shown to fold properly and exhibit antibody recognition akin to that of the native *Plasmodium* surface proteins (Dauvillée et al., 2010; Gregory et al., 2012; Jones et al., 2013). Algal chloroplast-produced *Pfs*25 was able to completely prevent malaria transmission, indicated by a total absence of *Plasmodium* oocysts in mosquito midguts after feeding on immunized mouse sera. Furthermore, feeding lyophilized algae expressing *Pfs*25 fused to CTB elicited a mucosal response to both antigens (Gregory et al., 2013). However, systemic IgG response was only observed for the CTB. This is in contrast with the *S. aureus* D2 protein fused to CTB, where systemic immunity was elicited for both domains (Dreesen et al., 2010), suggesting that either the furin protease cleavable linker between the *Pfs*25 and CTB domains prevented *Pfs*25 from being presented to the systemic immune system, or perhaps that *Pfs*25 is inherently less immunogenic. In a different strategy, truncated versions of the malarial proteins AMA1 and MSP1 were fused to the major protein constituent of the chloroplast starch granules, the granule-bound starch synthase (GBSS). Though they were expressed from the nuclear genome, reasonable accumulation was achieved because the proteins were targeted to and sequestered within the chloroplast starch granules. Both oral and injected vaccination using purified starch from these strains reduced parasite load and prolonged mice survival after challenge with *Plasmodium berghei*; in the case of an injected vaccine consisting of both antigens, 30% of mice survived the otherwise-lethal infection (Dauvillée et al., 2010).

All vaccines produced in algae to date are summarized in **Table 1**, along with reported yields and significant pre-clinical findings. Most work thus far has been performed in the green alga model organism *C. reinhardtii*, though one of the earliest reports of an algal-produced hepatitis B antigen was in the marine alga *Dunaliella salina *(Geng et al., 2003) and hepatitis B antigen has also been produced in the diatom *Phaeodactylum tricornutum *(Hempel et al., 2011)*. *In recent years the algal genetic toolkit has been expanded to other algal species, including other green algae, diatoms, and cyanobacteria (Ducat et al., 2011; Georgianna and Mayfield, 2012; Qin et al., 2012), with a goal of broad host range compatibility. Already, over 20 species of algae – including dinoflagellates, red algae, and diatoms – have been transformed, and a suite of promoters and selectable markers have been characterized for many species (see Gong et al., 2011, for a comprehensive review). While the first generation of algal vaccines has been predominantly pioneered in *Chlamydomonas, *these advances can readily be applied to alternative algal species that may be more suitable for large-scale vaccine production.

**Table 1:** Summary of algal-produced vaccines and significant findings.

Antigen(s)	Host/integration site	Yield	Significant findings	Citation
Foot-and-mouth disease virus (FMDV) structural protein VP1 fused to cholera toxin B subunit (CTB)	*C. reinhardtii* chloroplast genome	3 to 4% TSP	Protein accumulation was higher than reported in previous plant studies and detectable by Western and ELISA. Binding to GM1 ganglioside was weak but statistically significant. Strains were not completely homoplasmic, so higher yields may be possible.	Sun etal. (2003)
Hepatitis B surface antigen (HBsAg)	*D. salina* nuclear genome	Up to 3ng/mg soluble protein (0.0003% TSP)	Expression level was quantified by ELISA and reported to be “high” but the results reflect very low expression.	Geng etal. (2003)
Classical swine fever virus (CSFV) structural protein E2	*C. reinhardtii* chloroplast genome	1.5 to 2% TSP	Subcutaneous immunization of algal extracts induced systemic response, but oral immunization did not result in either systemic or mucosal response.	He etal. (2007)
Human glutamic acid decarboxylase 65 (hGAD65)	*C. reinhardtii* chloroplast genome**	0.25 to 0.3% TSP	Purified hGAD65 reacted with sera from diabetic mice, showing antigenicity. Higher yields may be obtained by codon-optimizing the human gene, which does not mimic the chloroplast AT content.	Wang etal. (2008)
White spot syndrome virus VP28 protein	*C. reinhardtii* chloroplast genome**	Variable, ranging from 0.2 to 20.9% total cellular protein (0.1 to 10.5% TSP)	The authors report high variability in expression levels, despite same-site integration by homologous recombination in the plastid genome. Stronger resistance to antibiotic selection might be useful for identifying high-expressing strains.	Surzycki etal. (2009)
*S. aureus* D2 fibronectin-binding domain fused to cholera toxin B subunit	*C. reinhardtii* chloroplast genome	Up to 0.7% TSP	Within whole cells, the antigen can withstand low pH and pepsin treatment, and is stable at room temperature for 20 months. Mice fed with whole algae showed mucosal IgA and systemic IgG responses to both CTB and D2, and 80% survived lethal *S. aureus* challenge.	Dreesen etal. (2010)
*P. falciparum* MSP1, *P. berghei* MSP1, and *P. berghei* AMA1, C-terminal domains only, fused to granule-bound starch synthase (GBSS)	*C. reinhardtii* nuclear genome, targeted to the chloroplast starch granules	0.2 to 1.0 μg of protein per mg of purified starch	The *P. falciparum* gene expressed significantly better than either *P. berghei* gene. A single dose was as effective as three doses in reducing parasite load after *P. berghei *challenge in vaccinated mice. Oral immunization with purified starch protected equally well as parenteral administration until day 40.	Dauvillée etal. (2010)
Hepatitis B surface antigen (HBsAg) fused to GFP or ER retention signal	*P. tricornutum *nuclear genome	0.7% TSP	By inhibitory ELISA, algal-produced HBsAg binds antibodies slightly stronger than commercially available HBsAg produced in yeast.	Hempel etal. (2011)
*P. falciparum* surface proteins *Pfs*25 and *Pfs*28	*C. reinhardtii* chloroplast genome	Not quantified; visible by Western and Coomassie after affinity purification	Conformation-specific antibodies and CD spectroscopy confirmed proper folding of heavily disulfide-bonded antigens. Sera from *Pfs*25-inoculated mice completely blocked replication within mosquitoes, and therefore malaria transmission.	Gregory etal. (2012)
*P. falciparum* surface protein *Pfs*25 fused to cholera toxin B subunit	*C. reinhardtii* chloroplast genome	Up to 0.09% TSP	Mice fed lyophilized algae showed mucosal IgA response to both CTB and *Pfs*25, but only systemic IgG response to CTB. A furin protease site separated the two domains.**	Gregory etal. (2013)
*P. falciparum* surface protein *Pfs*48/45 C-terminal domain	*C. reinhardtii* chloroplast genome	Not quantified; visible by Western blot after affinity purification	The antigenic C-terminal domain was recognized by Western blot and ELISA with conformation-specific antibodies. It appeared to accumulate in the insoluble fraction.	Jones etal. (2013)
Human Papillomavirus type 16 E7 protein, attenuated mutant (E7GGG)	*C. reinhardtii* chloroplast genome	Up to 0.12% TSP. His-tagged version obtained only 0.02% TSP.	In contrast to higher plant expression, *C. reinhardtii* obtained much higher yields and most of it was soluble. The crude algal extracts elicited lower IgG response than purified protein, but both preparations allowed 70% survival after tumor challenge.	Demurtas etal. (2013)
Angiotensin II fused to Hepatitis B virus capsid antigen (HBcAg)	*C. reinhardtii* nuclear genome	Up to 0.05% TSP	The chimeric antigen was visible by Western blot and quantitated by ELISA, but no* in vivo *or immunological activity assays were performed.	Soria-Guerra etal. (2014)

### FUTURE POTENTIAL FOR ALGAL-BASED ORAL RECOMBINANT VACCINES

From the research available to date, it is clear that algae can produce complex vaccine antigens, and that *Chlamydomonas*-produced antigens can elicit immunogenic responses that are appropriate for their intended roles as vaccines. It is also clear that identifying alternative mucosal adjuvants to complement these antigens is critical, whether for co-administration with algal-produced antigens or for incorporation into chimeric fusion proteins. It has been suggested that antigenic fusions with CTB, one of the preferred adjuvants, may interfere with the CTB subunit’s ability to form the pentameric structure essential for strong GM1 ganglioside binding (Sun et al., 2003). Many alternatives to CTB are under investigation for oral vaccination in other production platforms, including CpG-containing oligodeoxynucleotides, saponins, and subunits from heat-labile enterotoxin and ricin toxin (Pelosi et al., 2012). Future work should empirically explore many combinations of antigens, mucosal adjuvants, and even testing multiple linkers and potential translocation domains. As has been noted previously, expression, uptake, and antigenicity are all difficult to predict in the context of plant-produced oral vaccine antigens (Rybicki, 2009), so a high-throughput system like algae is extremely valuable for rapidly testing many versions of potential chimeric vaccine molecules. Furthermore, many antigens will require proper post-translational modifications such as glycosylation to be recognized properly; more work needs to be done to increase expression levels from the nuclear genome, as glycosylation does not occur in the chloroplast.

It has been suggested that the first licensed plant-produced human vaccines likely will not be the first ones tested in humans, many of which targeted pathogens like Hepatitis B for which a relatively inexpensive vaccine already exists (Rybicki, 2009). Stepping stones along the way to human vaccines may include reagents for cheaper diagnostics and development of veterinary vaccines. Several human studies with plant-made vaccines have also indicated a role for oral boosting of an existing immune response conferred by traditional injectable vaccines (Mason and Herbst-Kralovetz, 2012). An algal-produced human vaccine production platform will likely come to fruition as an alternative for very expensive vaccines like HPV, or for novel vaccines against diseases for which no alternative currently exists (Martinez et al., 2012). The cost and logistical considerations of storage, delivery, and administration in resource-limited settings indicate that plant or algal production may be the only feasible option for large-scale inexpensive vaccination, and thus this avenue deserves increased attention from research funding agencies and investment from the pharmaceutical industry as well.

## AUTHOR CONTRIBUTIONS

Elizabeth A. Specht and Stephen P. Mayfield wrote and revised the manuscript. Elizabeth A. Specht developed **Table 1**.

## Conflict of Interest Statement

The author Stephen P.Mayfield declares a financial interest in Triton Animal Health, a company making orally available nutritional supplements, and potentially orally available vaccines, should these prove to be biologically functional.
